# Triglyceride Levels Greater Than 10,000 mg/dL in a 49-Year-Old Female without Evidence of Pancreatitis

**DOI:** 10.1155/2019/6273196

**Published:** 2019-05-21

**Authors:** Anika Toor, Amit Toor, Koroush Khalighi, Mahesh Krishnamurthy

**Affiliations:** ^1^Department of Internal Medicine, Easton Hospital, Easton, PA 18045, USA; ^2^Drexel University, EP Lab, Easton Hospital, Easton, PA 18045, USA

## Abstract

We present a rare case of a 49-year-old female with very severe hypertriglyceridemia (HTG) having a total triglyceride (TG) count of > 10,000 mg/dL in the absence of pancreatitis. Based on literature review, this is one of the highest recorded TG counts in an adult without evidence of pancreatitis. HTG is a common occurrence in clinical practice, but rarely do numbers exceed 2000 mg/dl. It is crucial to evaluate and rapidly lower TG levels to prevent potentially life-threatening complications such as severe pancreatitis. Removal of potential predisposing medications, control of underlying diseases known to cause HTG, and maintenance therapies are essential to prevent reoccurrence.

## 1. Introduction

HTG is a common form of dyslipidemia with the potential to cause significant morbidity and mortality. In cases of severely elevated TG levels, rapid lowering to levels < 500–1000 mg/dL can be achieved with aggressive use of intravenous insulin and heparin (caution needs to be exercised when using heparin in cases of pancreatitis) and fasting.

## 2. Case Description

Our case is a 49-year-old Hispanic female who presented to our hospital with multiple episodes of chest pain. The onset of her pain was 5 days prior to admission. She complained of left sided pain “5 out of 10” intensity described as chest tightness. The pain was nonradiating and not precipitated by activity, inspiration, or position. The patient stated that she would have almost 5 episodes of pain daily with each episode lasting 2-5 minutes in length. This was not associated with any diaphoresis, shortness of breath, vomiting, or abdominal pain. The frequency and intensity of her pain episodes increased which led her to come to the emergency department. Her past medical history is significant for hypertension and difficult-to-control asthma requiring frequent hospital admissions. In the past she was on medication for “high cholesterol” but stopped taking it years ago as she was never told she had very high cholesterol levels. She denied any history of abnormal lipid profile, myocardial infarction or angina, congestive heart failure, or diabetes mellitus. Her medications included inhaled fluticasone and vilanterol combination, albuterol inhalation as needed, losartan, meloxicam, montelukast, verapamil, omalizumab, and intermittent short courses of prednisone for asthma exacerbations. She denied any alcohol consumption or illicit drug use. She admitted to smoking about a quarter pack a day for 15-20 years and quit 13 years ago. Family history was significant for hypertension in her mother, and a sister with stroke at the age of 44. There was no family history of dyslipidemia.

On physical examination she was found to have temperature of 97.8 degrees F, heart rate of 92 bpm, respiratory rate of 19/min with oxygen saturation of 96% on room air, and blood pressure of 152/98 mmHg. Her body mass index (BMI) was 27.7 on admission. Cardiovascular examination revealed chest wall tenderness to palpation on the upper left side. On auscultation there were no significant murmurs. Slight wheezing was audible in bilateral lung fields on auscultation. Examination of the skin did not reveal any xanthomas or xanthelasmas. Ophthalmologic examination did not reveal corneal arcus or lipemia retinalis. The examination of the nervous system and the head and neck was within normal limits.

**Figure 1:**
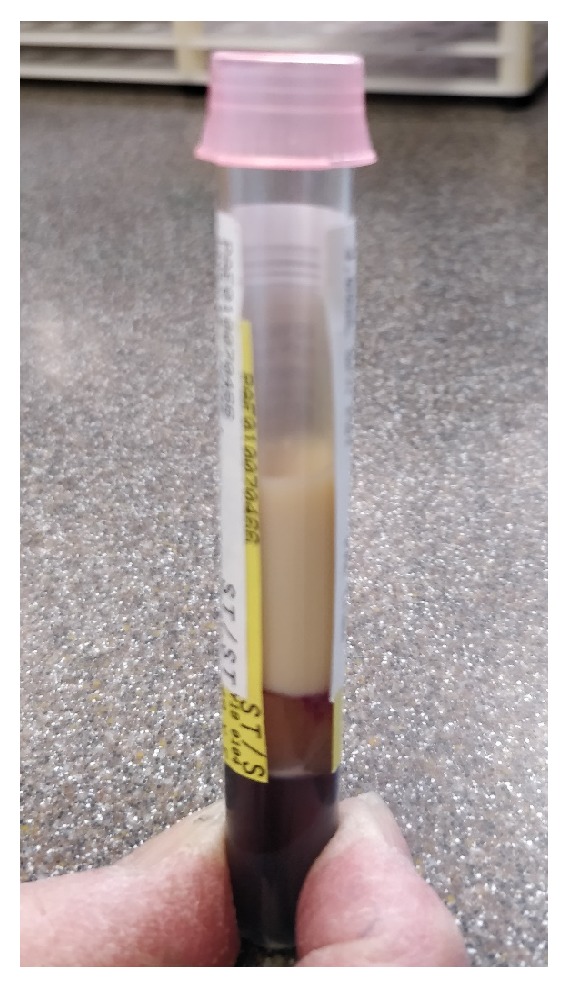


Relevant laboratory results on admission were as follows. Initial blood draw when centrifuged on gross examination was heavily lipemic (see Figure 1). Subsequent lipid panel studies were ordered which were significant for serum triglyceride (TG) of >10,000 mg/dL, serum cholesterol of 1,029 mg/dL, direct measure low density lipoprotein (LDL) of 33 mg/dL, and high-density lipoprotein (HDL) of 22 mg/dl. Other pertinent lab values included WBC count 7900/cumm, Hb of 11.6 g/dL, serum lipase of 160 units/dL, which was 46 units/dL when repeated and consistently remained within normal limits during hospitalization, serum sodium 130 mmol/L, serum magnesium of 5.2 mg/dL, and blood glucose of 111 mg/dL (random). Serum TSH was 2.39 mcIU/mL and HbA1c was of 5.3%. Initial troponin was of <0.03 ng/mL. Bicarbonate on admission was 22 mmol/L. EKG showed normal sinus rhythm with no ST-T wave changes. Chest X-ray was normal.

The patient was started on conservative management. She was kept nothing by mouth except medications for one day and started on IV regular insulin drip at a rate of 4 units/hr with IV fluids containing 5% dextrose to prevent hypoglycemia along with heparin 5000 units subcutaneously every 8 hours. Heparin drip was not initiated because PTT could not be accurately measured secondary to lipemia. She was also started on gemfibrozil, atorvastatin, and niacin at the time of admission. All of her home medications were stopped. A cardiac diet with no fats and no carbohydrates was initiated by day 2. Cardiology was consulted. Echocardiogram was done which was normal with an EF of 72%.

The patient's old medical record was reviewed to obtain previous lipid panel test results. Her lipid panel from 2016 revealed a serum triglyceride of 212 mg/dL and serum cholesterol of 182 mg/dL.

Lipid panel was repeated daily over the course of 9 days with significant decrease in patient's triglyceride and cholesterol levels. On day 9, serum triglyceride levels came down to 825 mg/dL and serum cholesterol was noted to be 360 mg/dL (see Table 1). Her diet was slowly advanced to a low fat and low carbohydrate diet which she tolerated well.

The patient also underwent treadmill myoview stress SPECT study once the serum triglyceride levels were < 2000 mg/dL in view of chest pain, which was normal. The patient's chest pain and serum triglyceride levels improved. She did not require any other interventions besides insulin drip and subcutaneous heparin. She was discharged on day 9 in medically stable condition and was closely monitored as outpatient. She was discharged on Rosuvastatin, Fenofibrate, and prescription omega-3 fatty acids. Repeat lipid panel was done in one month after discharge and the serum triglyceride levels were found to be 142 mg/dL and serum cholesterol of 117 mg/dL. Apolipoprotein B level was measured at 186 mg/dL. The patient was instructed for further genetic testing after discharge but did not follow up after this.

## 3. Discussion

Hypertriglyceridemia (HTG) is one of the most common lipid abnormalities and has been known to be associated with other metabolic derangements. It is estimated that the number of patients with HTG who have levels exceeding 500 mg/dL has risen to >4 million Americans and that this is more common in the Hispanic-American population [1, 2]. The presence of very severe HTG (TG levels greater than 2000 mg/dL) is estimated to be 1.8 cases per 10,000 white adults [3]. Hypertriglyceridemia is defined as TGs >150 mg/dL. The Endocrine Society 2010 guidelines further classify HTG into mild (150–199 mg/dL), moderate (200–999 mg/dL), severe (1000–1999 mg/dL), and very severe (greater than or equal to 2000 mg/dL) [4]. Severe and very severe HTG have been shown to increase the risk for acute pancreatitis and cardiovascular disease. When levels are > 1,000 mg/dL, the risk of pancreatitis rises to approximately 5%, and, when levels are >2000 mg/dl, the risk significantly rises to 10-20% [5].

The etiologies of HTG can be further classified into primary and secondary. The Fredrickson classification scheme is commonly used to organize various primary hypertriglyceridemias into different categories (Table 2). In the United States, the most common primary HTGs include familial combined hyperlipidemia (Fredrickson type IIb) and familial hypertriglyceridemia (Type IV) [6]. Secondary causes frequently include medical conditions such as obesity, untreated diabetes mellitus, excessive alcohol intake, hypothyroidism, nephrotic syndrome, liver disease, and pregnancy [4]. Certain medications are also well known to cause elevations of TG levels such as estrogens, glucocorticoids, thiazides, beta-blockers, antipsychotics (Olanzapine), immunosuppressants, protease inhibitors, isotretinoin, and bile acid resins [4, 6].

There is a broad spectrum of clinical manifestations of HTG, the most common of which being patients who are asymptomatic. Other symptoms may include mid-epigastric abdominal pain, nausea, and vomiting in relation to acute pancreatitis. Cutaneous manifestations are usually associated with elevations of LDL levels and include eruptive cutaneous xanthomas, palmar crease xanthomas, tendinous xanthomas, tuberous xanthomas, and eyelid xanthelasmas [7]. Ophthalmologic features can include lipemia retinalis. The risk of pancreatitis increases when TG levels exceed 1000-2000 mg/dL and is the third leading cause of pancreatitis after alcohol and gallstones. There are also moderate to highly significant associations between triglyceride values and the risk of coronary heart disease. We theorize that our patient experienced chest pain possibly as a result of accumulation of chylomicrons in the coronary arteries leading to ischemia which was relieved with rapid lowering of TG levels.

Diagnosis of specific HTG disorders requires extensive and costly workup. Therefore, the most critical initial step is to exclude secondary causes. This includes medication review, evaluation of body mass index (BMI), hemoglobin A1c, TSH, and renal and liver function testing [6]. When potential secondary causes have been ruled out, patients should be evaluated for various primary HTG.

Management of severe and very severe HTG involves rapidly lowering total TG levels to prevent development of acute pancreatitis. This comprises of both immediate dietary modifications and use of TG lowering medications which may require hospitalization [4]. During acute rapid lowering of TG levels, patients are typically kept NPO, and a no-fat diet is generally introduced when TG drops to <1000 mg/dL. For chronic maintenance, patients are instructed to avoid simple carbohydrates completely as well as reducing fat intake to less than 10% of daily caloric intake [8]. This is to decrease the risk of postprandial chylomicronemia since lipoprotein lipase (LPL) levels are maximally saturated from existing severe elevations of TG levels [9]. Rapid lowering of TG levels can be achieved with administration of IV Insulin drip with glucose and IV Heparin which are potent activators of LPL to effectively remove TGs from circulation [10, 11]. The effective goal to reduce the risk of pancreatitis is to reduce TG levels to less than 500-1000 mg/dL [4, 12–14]. Concomitantly, oral TG lowering medications should be initiated. First line medical therapy of isolated HTG is fibrates [6]. The preferred fibrate is Fenofibrate of which the onset of action may take a few weeks [4]. For patients with associated elevations of low-density lipoprotein (LDL) levels, a statin medication can be given. With this combination, risk of myopathy should be assessed with baseline creatinine kinase (CK) levels, and again if patients become symptomatic [15]. Suggested prescriptions of Omega-3-fatty acids and Niacin can be added as well. For patients who develop acute pancreatitis secondary to HTG, plasmapheresis can be performed to rapidly lower TG levels [16].

We postulate that our patient had a form of familial dyslipidemia which was exacerbated by the use of estrogens and steroids. Given her elevated apolipoprotein B levels, it is more likely that she had type IIb familial combined hyperlipidemia (FCHL).

## 4. Conclusion

Hypertriglyceridemia is frequently encountered in clinical practice. Very severe hypertriglyceridemia above 1000-2000 mg/dL should be evaluated for primary and secondary causes. Rapidly lowering triglyceride levels is essential to prevent serious complications such as acute pancreatitis. Long term effects of elevated TG count have also been associated with coronary artery disease. Rapid lowering can be achieved with intravenous insulin drip, even in nondiabetic patients, and intravenous heparin. Maintenance therapy with dietary changes and medications such as fibrates is highly recommended.

## Figures and Tables

**Table 1:** Patient's HTG trend along with therapies used and diet.

	Day 1	Day 2	Day 3	Day 4	Day 5	Day 6	Day 7	Day 8	Day 9
TG Levels (mg/dL)	>10,000	8,887	4,119	1,617	1,315	1,120	1,138	937	825
IV Drip	Insulin	Insulin	Insulin	Insulin	Insulin	Insulin	Insulin	Insulin	None
Diet	NPO	0 fat 0 carb	0 fat 0 carb	0 fat 0 carb	0 fat 0 carb	Low fat Low carb	Low fat Low carb	Low fat Low carb	Low fat Low carb
Oral Medication	G & A	G & A	G & A	G & A Niacin	G & A Niacin	G & A Niacin	G & A Niacin	G & A Niacin	F & R Niacin

G = Gemfibrozil; A = Atorvastatin; F = Fenofibrate; R = Rosuvastatin.

**Table 2:** Fredrickson classification of primary hypertriglyceridemia [6, 8].

TYPE	ELEVATED LIPOPROTEIN	LIPID PROFILE	CLINICAL MANIFESTATIONS	RELATIVE FREQUENCY
I – Familial chylomicronemia	Chylomicron	TC + TG +++	Presents in infancy, eruptive xanthomas, recurrent pancreatitis, failure to thrive	<1%

II b – Familial Combined Hyperlipidemia	LDL, VLDL, Apo-B	TC ++ TG ++	Xanthomas less common. Risk of premature CVD	40%

III – Familial dysbetalipoproteinemia	IDL	TC ++ TG ++	Palmar xanthomas, risk of premature CVD	<1%

IV – Familial Hypertriglyceridemia	VLDL	TC + TG ++	Associated w/ DM, insulin resistance, obesity, HTN	45%

V – Primary Mixed Hyperlipidemia	Chylomicron, VLDL	TC +++ TG +++	Similar to type I but develops in adulthood	5%

TC = total cholesterol; TG = triglycerides; DM = Diabetes Mellitus; HTN = Hypertension; CVD = cardiovascular disease; LDL = low-density lipoprotein; VLDL = very low-density lipoprotein; IDL = intermediate density lipoprotein; Apo-B = apolipoprotein B.
